# A sandwich enzyme-linked immunosorbent assay for the quantitation of human plasma ferritin

**DOI:** 10.1016/j.mex.2018.06.010

**Published:** 2018-06-18

**Authors:** Andie V. Bleicher, Holger W. Unger, Stephen J. Rogerson, Elizabeth H. Aitken

**Affiliations:** a Department of Medicine at the Doherty Institute, University of Melbourne, Melbourne, Australia; b Department of Obstetrics and Gynaecology, Royal Infirmary of Edinburgh, Edinburgh, UK

**Keywords:** Measurement of ferritin by ELISA, ELISA, Ferritin, Anemia, Iron deficiency, Plasma

## Abstract

There is a lack of published enzyme linked immunosorbent assay (ELISA) protocols which use commercially available reagents for the measurement of ferritin in human plasma for research purposes. ELISA kits are often expensive and do not always provide detailed information about reagents included. A commercially available antibody pair was used to develop an in-house ELISA to measure ferritin in small (25 μl) volumes of human plasma. ELISA results were compared to ferritin levels measured using a commercial immune-assay system. The sensitivity, intra and inter assay variation of the ELISA assay were also determined. ELISA measurements of plasma ferritin ranged between 3.2–232 ng/mL and were comparable to those measured by a commercial immunoassay system (Pearson correlation r = 0.93 P < 0.0001). Ferritin levels as low as 0.5 ng/mL were detectable and samples with both low and normal ferritin levels showed low inter and intra-assay variation. This ELISA protocol allows the accurate, reliable and cost-effective quantitative determination of plasma ferritin levels from small volumes of human plasma.

• No published protocols detail how to measure ferritin by ELISA using commercially available antibodies.
• ELISA kits are expensive and information on antibodies included are often lacking.
• We have identified a commercially available antibody pair to measure plasma ferritin using a cost-effective ELISA.

## Graphical abstract

**Figure:**
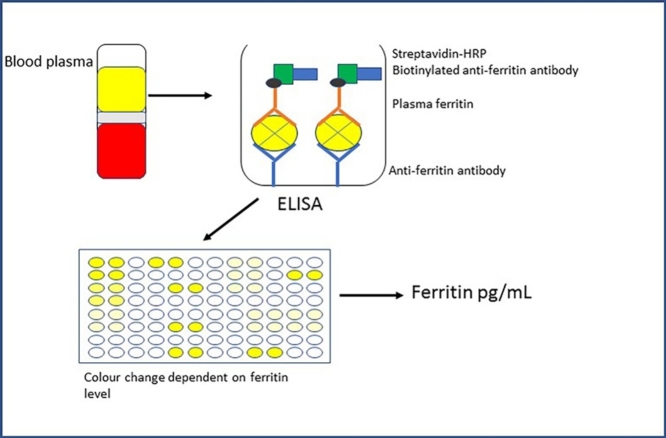

**Specifications Table**

**Table:** 

Subject area	*Select one of the following subject areas*:
	• *Medicine and Dentistry*
More specific subject area	*Iron deficiency*
Method name	*Measurement of ferritin by ELISA*
Name and reference of original method	**Name:** Serum ferritin by a rapid and inexpensive ELISA method. **Reference:** Anderson MG, Kelly AM. Serum ferritin by a rapid and inexpensive ELISA method. Clin Chim Acta. 1981 Nov 11;116(3):405-8.
Resource availability	

## Method details

### Background

Plasma ferritin is a marker of iron stores and along with haemoglobin concentration, it can be used to identify iron deficiency anemia [1,2], a condition with a large health burden, contributing to maternal and perinatal mortality as well as maternal cognitive impairment and reduced fitness and productivity [3]. The Global Burden of Disease 2000 project estimated that iron deficiency anemia accounts for 841 000 deaths and 35 million disability-adjusted life years [3].

There are published protocols for in-house enzyme linked immunosorbent assays (ELISAs) to measure whole ferritin in plasma [4,5], but the antibody pair described in these protocols is no longer commercially available. With this in mind we have identified a new antibody pair which we then used to develop a cost-effective ELISA to measure plasma ferritin for research purposes.

### Protocol

To do the ELISA Nunc MaxiSorp® flat-bottom 96 well plates (ThermoFisher Scientific 442404) were coated with 100 μl/well goat anti-ferritin polyclonal antibody (Abcam ab33574) diluted to 367 ng/mL in phosphate buffered saline (PBS), overnight at 4 °C. The following day, the plates were washed with wash buffer (0.05% Tween20® in PBS) and then blocked with 200 μl/well of reagent diluent (1% Bovine Serum Albumin (Sigma A7906), 0.05% Tween20® in PBS) for 1 h at room temperature. After further washing, 100 μl/well of samples (human plasma diluted 1 in 8 in reagent diluent) and standards (Liquichek Immunology Control L3 (BIORAD 596) diluted 1 in 4 in reagent diluent followed by a 1 in 2 serial dilution over 10 points) were aliquoted in duplicate and incubated for 2 h at room temperature. After further washing, 100 μl of biotinylated rabbit anti-ferritin polyclonal antibody (Abcam ab7333) diluted to 125 ng/ml in reagent diluent was added to each well for 1 h at room temperature, plates were washed, and 100 μl of streptavidin-horseradish peroxidase (Abcam ab7403) diluted to 67 ng/mL in reagent diluent was added to each well for 1 h at room temperature. Plates were washed and 100 μl of 3,3′,5,5′-tetramethylbenzidine substrate (BD 555214) was added to each well. After 30 min at room temperature 50 μl/well of stop solution (1 M H_2_SO_4_) was added. The optical density at 450 nm was measured using a FLUOstar Omega BMG LABTECH microplate reader. A standard curve was generated and values for unknown samples were extrapolated.

### Development and validation methods

Working concentrations of capture and detection antibodies were determined by a series of checkerboard dilutions using a pool of plasma as a sample (capture and detection antibodies were tested at concentrations ranging from 0.2 to 11 and 0.125–5 μg/mL respectively). Selected concentrations chosen based on results from checkerboard dilutions were further tested using a ferritin standard curve (Liquichek Immunology Control L3 (BIORAD 596)) and three test plasma samples of known ferritin concentrations (13 ng/mL, 27 ng/mL and 54 ng/mL ferritin). Working concentrations were finally decided based on the lowest capture and detection antibody concentrations which gave a clear standard curve and which allowed the accurate determination of ferritin concentration in the three test samples.

To validate the method seven standards with ferritin concentrations of 0.88-0.028 ng/mL were measured in 10 individual assays and compared to a no-ferritin control sample to determine assay sensitivity. Twenty one plasma were assayed by Melbourne Health Shared Pathology Service at the Royal Melbourne Hospital using the commercial immunoassay system-the ARCHITECT Ferritin assay, a certified diagnostic two-step chemiluminescent microparticle immunoassay [6]. Results from these samples were compared to results from the in-house ELISA.

Two plasma samples with known ferritin concentrations, (one low and one normal) were included as comparators in 17 individual assays carried out over a period of 4 months to confirm that the ELISA provided consistent results. Two additional samples were measured 6 times within one assay to compare intra-assay variation.

### Development and validation results

**Fig. 1:**
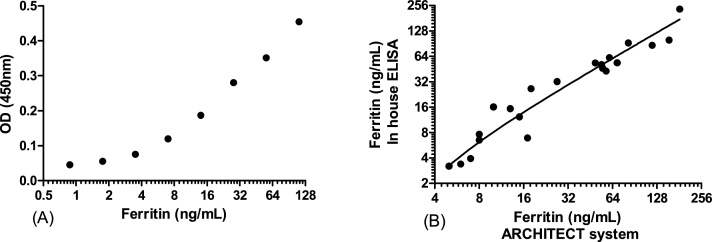
**In house ferritin ELISA accuracy**. (A) Example of a standard curve from the in house ELISA (B) Comparison of the in house ELISA with the Architect system (Abbott) a commercial immunoassay, Pearson correlation r = 0.93 P < 0.0001, n = 21.

Working concentrations of capture and detection antibody and the range of standards used in experiments resulted in a clear standard curve (see example in Fig. 1a) and gave results for three test samples (15, 28 and 47 ng/mL ferritin) similar to their known concentrations (13 ng/mL, 27 ng/mL and 54 ng/mL ferritin respectively).

Ferritin dilutions of 0.88 and 0.44 ng/mL gave readings higher than those of the no ferritin control in 10 out of 10 plates. Ferritin dilutions of 0.22 ng/mL were higher than the no ferritin control in only 3 out of 10 plates, ferritin dilutions of 0.11 ng/mL and lower were detectable in 0 out of 10 plates. Plasma ferritin values in 21 samples using in house ELISA ranged between 3.2–232 ng/mL and comparison of ferritin levels using in house ELISA and those measured by the ARCHITECT system at the Melbourne Health Shared Pathology Service show that the two assays give very similar results (Fig. 1b). In addition, the proportion of individuals identified as suffering iron deficiency anaemia (ferritin <15 ng/mL [1]) using the in-house ELISA was the same as those identified using the commercial immunoassay system-the ARCHITECT Ferritin assay [6] (7 out of 21 individuals).

Repeated measurement of two plasma samples over individual assays to assess inter-assay variation showed inter-assay consistency and accuracy with samples of both low ferritin (13 ng/mL) and normal ferritin (54 ng/mL) (coefficient of variation 27.8% and 25.7% respectively) (Table 1) and measurements of another two plasma samples repetitively within a single plate showed intra-assay results are also consistent (intra-assay coefficients of variation were 7% and 13.7%) (Table 1).

**Table 1:** In house ferritin ELISA reproducibility.

	Actual (ng/mL)^a^	mean(ng/mL)	SD	CV (%)^c^
	Inter assay variation b			
Sample 1	13	14.5	4	27.8
Sample 2	54	58.5	15	25.7
	Intra assay variation d			
Sample 3		40.7	2.8	7.0
Sample 4		30.3	4.2	13.7

a Levels as measured in a single assay by ARCHITECT system.

b Values from 17 different assays carried out over a period of 4 months.

c CV, coefficient of variation.

d Values from 6 repeats of samples on a single plate.

This human ferritin ELISA protocol allows simple, convenient and low-cost measurements of ferritin in human plasma for research purposes. It provides results which correlated well with those of a commercial immunoassay system, including at concentrations below 30 ng/mL which is the range of interest for those looking at iron deficiency anemia [1]. The sensitivity of around 0.5 ng/mL is sufficient to measure ferritin even in individuals who have very low levels and is comparable to the Abbot Architect immunoassay system (<1 ng/mL) [6]. The assay we describe is substantially cheaper than commercially available ELISA kits, with reagent costs of this inhouse ELISA at approximately US$23/plate compared to US$350–450/plate for commercial kits at 2018 prices. Advantages over a commercial immunoassay system include that it can be done with minimal equipment, high throughput formats can be established, and it is cost-efficient. Our local charge for outsourcing plasma ferritin measurements for measurement using a commercial immunoassay system was a minimum of US$8.00/sample at 2018 prices. The nature of the antibodies used is fully disclosed, as is data on the isoform/s of ferritin the antibodies recognise. The antibodies used in this ELISA are polyclonal and raised against full length native human liver ferritin. Ferritin is a 24 subunit-protein comprised of two types of subunits (H and L) and the ratio of the different subunits varies with the ferritin isoform, for example ferritin from the heart contains mostly the H subunit but ferritin from the liver (and also ferritin in plasma) is primarily made up of the L subunit (reviewed in [7]). It has been suggested that variation in the isoform being measured is one possible explanation of variation between different assay systems for ferritin measurement [8] and therefore information regarding the source of ferritin towards which the antibodies used in assays were generated is important for comparing results measured using different methods.
